# Self-perceived symptoms and care needs of patients with severe to very severe chronic obstructive pulmonary disease, congestive heart failure or chronic renal failure and its consequences for their closest relatives: the research protocol

**DOI:** 10.1186/1472-684X-7-5

**Published:** 2008-05-06

**Authors:** Daisy JA Janssen, Emiel FM Wouters, Jos MGA Schols, Martijn A Spruit

**Affiliations:** 1 Central Department of Treatment and Care, Proteion Thuis, Horn, The Netherlands; 2 Department of Respiratory Medicine, University Hospital Maastricht, Maastricht, The Netherlands; 3 Director, Centre for Integrated Rehabilitation of Organ failure (CIRO), Horn, The Netherlands; 4 Department of General Practice, Faculty of Health Medicine and Life sciences, University Maastricht, Maastricht, The Netherlands; 5 Department of Research, Development & Education, Centre for Integrated Rehabilitation of Organ failure (CIRO), Horn, The Netherlands

## Abstract

**Background:**

Recent research shows that the prevalence of patients with very severe chronic obstructive pulmonary disease (COPD), congestive heart failure (CHF) and chronic renal failure (CRF) continues to rise over the next years. Scientific studies concerning self-perceived symptoms and care needs in patients with severe to very severe COPD, CHF and CRF are scarce.

Consequently, it will be difficult to develop an optimal patient-centred palliative care program for patients with end-stage COPD, CHF or CRF. The present study has been designed to assess the symptoms, care needs, end-of-life care treatment preferences and communication needs of patients with severe to very severe COPD, CHF or CRF. Additionally, family distress and care giving burden of relatives of these patients will be assessed.

**Methods/design:**

A cross-sectional comparative and prospective longitudinal study in patients with end-stage COPD, CHF or CRF has been designed. Patients will be recruited by their treating physician specialist. Patients and their closest relatives will be visited at baseline and every 4 months after baseline for a period of 12 months. The following outcomes will be assessed during home visits: self-perceived symptoms and care needs; daily physical functioning; general health status; end-of-life care treatment preferences; end-of-life care communication and care-giver burden of family caregivers. Additionally, end-of-life care communication and prognosis of survival will be assessed with the physician primarily responsible for the management of the chronic organ failure. Finally, if patients decease during the study period, the baseline preferences with regard to life-sustaining treatments will be compared with the real end-of-life care.

**Discussion:**

To date, the symptoms, care needs, caregiver burden, end-of-life care treatment preferences and communication needs of patients with very severe COPD, CHF or CRF remain unknown. The present study will increase the knowledge about the self-perceived symptoms, care-needs, caregiver burden, end-of-life care treatment preferences and communication needs from the views of patients, their loved ones and their treating physician. This knowledge is necessary to optimize palliative care for patients with COPD, CHF or CRF. Here, the design of the present study has been described. A preliminary analysis of the possible strengths, weaknesses and clinical consequences is outlined.

## Background

Chronic diseases like chronic obstructive pulmonary disease (COPD), congestive heart failure (CHF) or chronic renal failure (CRF) are nowadays major causes of morbidity and mortality worldwide.[1] Recent research shows that the prevalence of patients with very severe COPD [2], CHF [3] and CRF [4] continues to rise over the next years.

Life prolongation is not the ultimate goal of management of patients with severe to very severe COPD, CHF or CRF. Management of these patients should prevent, relieve and/or soothe self-perceived symptoms and care needs to optimise daily functioning and stabilize disease-specific health status. [5-9] The management of patients with severe to very severe COPD, CHF or CRF should probably be a holistic type of care, equally concerned with physical, psychosocial and spiritual aspects of each patient.[10]

To the best of the present authors' knowledge, no management/treating program is currently aiming at the self-perceived symptoms and care needs of patients with severe to very severe COPD, CHF or CRF.

In fact, it is currently unknown whether and to what extent patients with severe to very severe COPD, CHF or CRF suffer from self-perceived symptoms before they enter the last phase of their life and how they develop throughout the final course of the disease. At present, it is only known that patients with end-stage COPD, CHF or CRF have suffered from incapacitating symptoms while only a minority of these symptoms/problems has been treated appropriately. [11-16] In the interest of patients with severe to very severe COPD, CHF or CRF, but also in the interest of their close relatives, it is of major importance to extent the currently available knowledge concerning assessment and treatment of self-perceived symptoms and care needs to optimise existing treatment options, to develop completely new treatment options and to offer adequate relief of self-perceived symptoms and care needs.

It remains very difficult to predict survival in patients with end-stage chronic organ failure. Indeed, physicians' predictions of survival in patients with various serious chronic conditions were often erroneous and optimistic.[17,18] Moreover, a fundamental issue affecting the prediction of survival of patients with severe to very severe COPD or CHF is the hypothetical death trajectory: a slow decline in quality of life punctuated by acute exacerbations of COPD or CHF from which patients with severe to very severe COPD or CHF will only partially recover until a final crisis occurs that cannot or will not be treated. [19-21]

It is therefore of great importance for patients with severe to very severe COPD, CHF or CRF to get to know and discuss survival and the preferences and consequences of life-sustaining interventions (i.e. mechanical ventilation and cardiac resuscitation) with their family and the treating physician.[22] In fact, finding out and honouring the treatment preferences of terminally ill patients is most probably critical for the provision of high-quality end-of-life care. Moreover, patients with severe to very severe COPD, CHF or CRF who want to spend their end-of-life period as they want, should probably leave better advance directives. For example, a recent study in patients with chronic renal failure reported that about one-third of the close relatives and about one-third of the treating physicians predicted incorrectly the current preferences for cardiopulmonary resuscitation and haemodialysis under various circumstances.[23] The contents and quality of end-of-life communication between patients with severe to very severe COPD, CHF or CRF, their closest relatives and their physician remain currently unknown.

Patients with severe to very severe chronic diseases like COPD, CHF or CRF may experience problems with performing normal daily tasks.[24] When the patient is still living at home, most of the care needs may be carried out by family members.[25] This may involve participation in personal hygiene needs, administration of medication, attention to nutritional needs, psychological support and emergency management of problems such as excessive shortness of breath [26], but also heavy physical work of transferring a weak patient and attending needs such as laundering and cleaning. A recent study in 18 patients with COPD, CHF or CRF suggests that decreased independence and social isolation imposes a considerable burden of care on the family.[10] Additionally, Barnes and colleagues have shown that carers of CHF patients have other characteristics than carers of patients with cancer and need practical and emotional support.[27] At present, it remains unknown whether and to what extent self-perceived symptoms and care needs of patients with severe to very severe COPD, CHF or CRF may result in an extra caregiver burden for close relatives. It is therefore reasonable to hypothesize that for the families and loved ones of patients with severe to very severe COPD, CHF or CRF there is also great distress, which may increase while the symptoms and care needs of patients with severe to very severe COPD, CHF or CRF progress over time. Indeed, standing witness of physical [28] and emotional distress of the patient [29], bearing the burdens of extra care [25] and future anticipated loss will most probably cause substantial family distress. By identifying, understanding and addressing factors that may potentially overcome patients with severe to very severe COPD and their family, the necessary preconditions for coping and managing of will be established.[6]

Currently, the change over time in self-perceived symptoms, care needs and preferences with regard to life-sustaining treatments and communication about end-of-life care in patients with severe to very severe COPD, CHF or CRF remain unknown too.

### Aims of the study

In the present study we aim to elaborate the currently available knowledge concerning the self-perceived symptoms, care needs, caregiver burden, preferences with regard to life-sustaining treatments and communication about end-of-life care in patients with end-stage chronic organ failure, in particular COPD, CHF or CRF. To achieve this aim we need to answer the following questions:

1.1 What are the self-perceived symptoms and care needs of patients with severe to very severe COPD, CHF or CRF and to what extent do they differ from each other?

1.2 Do these self-perceived symptoms and care needs relate to patients' daily functioning, health status and mood status?

1.3 Whether and to what extent are self-perceived symptoms and care needs different between patients with severe COPD, very severe COPD without long-term oxygen therapy or very severe COPD with long-term oxygen therapy?

2.1 How are the self-perceived symptoms of patients with severe to very severe COPD, CHF or CRF perceived by their closest relatives?

2.2 Whether and to what extent do self-perceived symptoms and care needs of patients with severe to very severe COPD, CHF or CRF affect caregiver burden?

3.1 What are the preferences with regard to life-sustaining treatments of patients with severe to very severe COPD?

3.2 Whether and to what extent do the preferences with regard to life-sustaining treatments of patients with severe to very severe COPD differ from those of patients with end-stage CHF or CRF?

4.1 How do patients with severe to very severe COPD, CHF or CRF and their closest relatives perceive end-of-life care planning (i.e. communication about survival prognosis and life-sustaining treatments) with the treating physician?

5.1 How do self-perceived symptoms, care needs, caregiver burden, preferences with regard to life-sustaining treatments, end-of-life care communication and management of patients with severe to very severe COPD, CHF and CRF change over time according to the patients and their closest relatives?

The objective of this article is to describe the design of the present study and inform others on the possibilities of performing end-of-life care research in end-stage chronic organ failure, especially in COPD, CHF or CRF. In particular, the prospective and longitudinal study design, in which the views of patients, their relatives and physician will be taken into account for the assessment of daily symptoms, care needs, caregiver burden, end-of-life care treatment preferences and communication needs in three groups of patients with end-stage chronic organ failure has not been described before. The described methodology will also serve as detailed reference for the method section of future publications of this study.

## Methods/design

### Design

**Figure 1:**
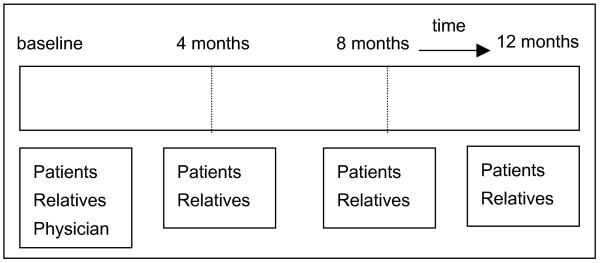
**Study design**. Timing of the interviews.

A cross-sectional comparative study in patients with end-stage COPD, CHF or CRF has been designed to answer question 1.1 to 4.1. Additionally, a longitudinal follow-up study has been designed to answer question 5.1. Patients and their closest relatives will be visited at baseline and every 4 months after baseline for a period of 12 months. Figure 1

### Study population

The study population consists of patients with severe to very severe COPD, end-stage CHF and end-stage CRF. All patients will be recruited by their physician specialist (n = about 25) at the outpatient consultation in one academic hospital and six general hospitals throughout the southern-eastern part of the Netherlands. Patients and their closest relatives will be included. Additionally, the participating physician specialist primarily responsible for the management of the chronic organ disease of the enrolled patients will be included.

Inclusion criteria are: patients with severe COPD (Global initiative for chronic Obstructive Lung Disease (GOLD) classification III); patients with end-stage COPD (GOLD classification IV) without long-term oxygen therapy (LTOT); patients with end-stage COPD (GOLD classification IV) with LTOT; patients with end-stage CHF (New York Heart Association (NYHA) classification III and IV) and patients with end-stage CRF (requiring dialysis).

Exclusion criteria are: the patient is not clinically stable for at least 4 weeks preceding enrolment (no hospital admission or major change in medication, according to the treating physician specialist); pharmacological therapy is not optimal (according to the current available guidelines) and stable for at least 2 months preceding enrolment and patients in a nursing home.

The lack of previous results makes it impossible to perform power calculations. The intended sample size will be 150 patients with COPD (GOLD classification III, n = 50; GOLD classification IV without LTOT, n = 50; GOLD classification IV with LTOT, n = 50); 100 patients with CHF (NYHA class III, n = 50; NYHA class IV, n = 50) and 100 patients with CRF (haemodialysis, n = 50; peritoneal dialysis, n = 50).

### Instruments

After oral and written informed consent has been given by participating patients, the following outcomes will be assessed at the patients' home-environment: demographics; current self-reported comorbidities: Charlson comorbidity index [30]; disease history; general health status: EuroQol-5 Dimensions (EQ-5D) [31], Assessment of Quality Of Life (AQOL) [32], Medical Outcomes Study 36-Item Short-Form Health Survey (SF-36) [33]; disease-specific health status: St. Georges Respiratory Questionnaire (SGRQ, only for COPD patients) [34], Minnesota Living with Heart Failure Questionnaire (MLHFQ, only for CHF patients) [35], Kidney Disease Quality Of Life questionnaire (KDQOL, only for CRF patients) [36]; anxiety and depression: Hospital Anxiety and Depression Scale (HADS) [37]; daily physical functioning: Timed 'Up and Go' test (TUG) [38], Care Dependency Scale (CDS) [39]; symptom checklist for the patients to determine the degree of self-perceived physical and psychological symptoms using Visual Analogue Scales (VAS); current disease management checklist; end-of-life care communication: Quality Of Communication questionnaire (QOC) [40], Barriers and Facilitators Questionnaire (BFQ) [41]; end-of-life care treatment preferences [42], Willingness to Accept Life-sustaining Treatments questionnaire (WALT) [22]; weight and length (only at baseline). Additionally, NT-proBNP [43], creatinine and lung function (spirometry, Forced Expiratory Volume in the first second (FEV1)) will be measured at baseline to assess previously unrecognised co-existing morbidities.

Patients will be asked to identify the person who spends most time with them and provides most of their care, assistance and support.[44] After oral and written informed consent has been given by participating close relatives of enrolled patients, the following outcomes will be assessed at their home-environment: care-giver burden: Family Appraisal of Care giving Questionnaire for Palliative Care (FACQ-PC) [45]; perception of the patient's symptoms checklist; barriers and facilitators in end-of-life care communication: BFQ [41] and general well being: semi-structured interview. If possible, relatives will not be interviewed in the presence of the patient.

The participating physician specialist primarily responsible for the management of the chronic organ disease of the enrolled patients will be interviewed at baseline to assess end-of-life care communication and prognosis of survival of the patient with chronic organ disease.

If patients decease during this period, the first author will call the relatives to compare the most recent end-of-life care treatment preferences of the patient with the real end-of-life care (e.g. place of dying, the use of life-sustaining treatments like resuscitation or invasive ventilation).

Finally, for patients with COPD or CHF the number of exacerbations/acute heart decompensation during every 4-month period will be assessed using a diary, which has to be filled out every day. Complications of dialysis will be assessed every four months at the dialysis department.

From a group of non-participating patients some data like severity of disease (GOLD classification or NYHA classification), age and gender will be collected.

Questionnaires that were not available in Dutch (AQOL, QOC, BFQ, WALT and FACQ-PC) have been translated into Dutch by the procedure of forward-backward translation.

### Ethical considerations

The study will be conducted according to the principles of the Declaration of Helsinki [46] and in accordance with the Medical Research Involving Human Subjects Act (WMO).

The load for patients has been tested in three volunteering patients with end-stage COPD and two relatives. The load for patients will be low, because patients will be visited at home, mainly for filling in questionnaires. This will probably not increase discomfort and sensations like dyspnea.

All patients will be recruited at the outpatient consultation of their treating physician in one academic hospital and six general hospitals. Patients will receive the patient information letter and the informed consent form. Patients will be informed verbally by a research nurse. Patients will be asked for their consent by the research nurse after a week. If patients can not decide about their participation at that moment, the research nurse will call again after another week. Data will be handled confidentially and anonymously. The protocol of the present study has been approved by the Medical Ethical Committee of the University Hospital Maastricht (NL16264.068.07/MEC 07-3-054).

### Data management and plan of statistical analysis

Data are entered weekly in a database. Missing data will be minimized because patients will be visited at home for filling in the questionnaires and a research nurse will check if all the questions have been answered. Handling of missing data will be done according to the guidelines of the different questionnaires. For data-analysis SPSS 15.0 will be used. All operations will be stored in a SPSS-syntax file. Interim analysis will be done when each group consists of 50 patients.

Categorical variables will be described as frequencies, while continuous variables will be tested for normality and will be presented as mean and standard error of the mean (SEM) or median, interquartile range and minimum and maximum. Comparison of baseline results between patients with COPD, CHF and CRF will be done using parametric and non-parametric statistics and multivariate analysis, as appropriate. The presence of co-existing morbidities will be taken into account. To estimate longitudinal changes, a mixed effect model for the slopes can be used. In these analyses, covariates such as age, gender and smoking status can be included as fixed effects, whereas time can be entered as random effect. Bivariate relationships between slope changes will be analysed by using a Pearson's correlation coefficient. Comparison baseline results between surviving patients with patients who passed away during the 1-year follow up period will be done using parametric and non-parametric statistics, as appropriate. A priori, a two-sided level of significance will be set at p ≤ 0.05.[47] Additionally, ninety-five percent confidence intervals will be provided to assist in determining the clinical significance of the differences of results between groups of patients.

## Discussion

The present study has been designed to assess the self-perceived symptoms, care needs, caregiver burden, preferences with regard to life-sustaining treatments and communication about end-of-life care in patients with end-stage chronic organ failure, in particular COPD, CHF or CRF. Patients with moderate to severe COPD, CHF or CRF have been shown to suffer from exercise intolerance, muscle weakness and abnormal changes in body composition, irrespective of the degree of primary organ failure. [48-51] Therefore, it seems reasonable to hypothesize that patients with end-stage COPD, CHF or CRF have comparable daily symptom burden and care needs.

This study has several strengths and weaknesses, which will be described below.

### Strengths

The present study concerns patients with end-stage chronic, non-malignant, diseases: COPD, CHF and CRF. To the present authors' knowledge, this study is the first to assess symptoms, care-needs, caregiver burden, end-of-life care treatment preferences and communication needs from the views of patients with severe to very severe COPD, patients with end-stage CHF and patients with end-stage CRF, their loved ones and their treating physician. Previously have been described the methodological challenges of research in end-of-life care, like the difficult task of prospectively identifying the terminally ill.[52] To date, many studies concerning end-of-life care have a cross-sectional and retrospective design. Barnes and colleagues have previously described the importance of prospective and longitudinal research in chronic diseases.[53] The prospective and longitudinal design of this study will allow studying end-of-life care in patients who are not previously identified as terminally ill. Moreover, the longitudinal and prospective design is less vulnerable for measurement error, which is a risk of cross-sectional or retrospective studies.[54,55] The intended sample size and the involvement of multiple hospitals (six general and one academic hospital) will improve the reliability of the results. The present study will provide valuable data to an area that has only scarcely been studied before.

### Weaknesses

Patients will be recruited by their treating physician specialist at the outpatient consultation. The external validity of the results of the study may be limited due to the exclusion criteria. For example, patients who are not clinically stable for four weeks will not be included. However, the exclusion of patients who are not clinically stable has the major advantage that all patients are clinically stable at baseline which improves the internal validity of the study.

Additionally, it is possible that participating patients have other clinical characteristics than non-participating patients. For example, it is possible that participating patients will be patients who talk more easily about end-of-life care than patients who do not want to take part in the study. Moreover, it is possible that physicians do not want to ask their terminally ill patients to participate in the study.

Nevertheless, the risk for selection bias will be reduced by the participation of seven hospitals and about 25 physicians in the recruitment of patients. Additionally, we will collect some data from a group of non-participating patients as mentioned before.

A part of the included patients will probably have co-existing morbidities, not included in the Charlson comorbidity index, like osteoarthritis, which could possibly affect symptom experience, care needs, health status and caregiver burden. Therefore, patients will be asked to report co-existing morbidities not included in the Charlson comorbidity index.

The lack of previous results makes it impossible to perform power calculations. Therefore a lack of power of this study may be possible.

Finally, previous authors described the challenges of performing prospective longitudinal research in patients with end-stage chronic diseases.[44,53] However, the recruitment in the present study will be stimulated by the involvement of about 25 physicians of 7 hospitals and the active role of the research nurses in recruitment. The home visits by a research nurse or researcher will stimulate sustaining participation of patients and caregivers. This will provide patients and caregivers the most convenient setting for data collection.

### Clinical consequences

The present study will identify the daily symptoms experienced by patients with severe to very severe COPD, CHF and CRF. The present study will identify the care needs which should receive attention in the development of management programs for patients with severe to very severe COPD, CHF or CRF. Additionally, the present study will reveal the end-of-life care treatment preferences and, if present, the changes in treatment preferences of patients with end-stage COPD, CHF or CRF, which will identify which and when treatment preferences should be discussed with the individual patient and his or her loved ones. The communication needs and barriers and facilitators of end-of-life care communication which will be found in this study may give recommendations for improvement of end-of-life care communication between patients with end-stage COPD, CHF or CRF, their loved ones and treating physician. Finally, the present study will identify the factors affecting caregiver burden, which may increase the possibilities to influence caregiver burden in future management programs.

## Conclusion

To date, the symptoms, care needs, caregiver burden, end-of-life care treatment preferences and end-of-life care communication of patients with very severe COPD, CHF or CRF have been studied scarcely. The present study will increase the knowledge about the symptoms, care needs, caregiver burden, end-of-life care treatment preferences and communication needs from the views of patients, their families and their treating physician. This knowledge is necessary to optimize palliative care for patients with COPD, CHF or CRF. Here, the study protocol is described and a preliminary analysis of the possible strengths, weaknesses and clinical consequences is outlined.

## Abbreviations

AQOL: Assessment of Quality Of Life; BFQ : Barriers and Facilitators Questionnaire; CDS: Care Dependency Scale; CHF: Chronic Heart Failure; COPD: Chronic Obstructive Pulmonary Disease; CRF: Chronic Renal Failure; EQ5D: EuroQol-5 Dimensions; FACQ-PC: Family Appraisal of Care giving Questionnaire for Palliative Care; FEV1: Forced Expiratory Volume in the first second; GOLD: Global initiative for chronic Obstructive Lung Disease; HADS: Hospital Anxiety and Depression Scale; KDQOL: Kidney Disease Quality Of Life questionnaire; LTOT: Long-Term Oxygen Therapy; MLHFQ: Minnesota Living with Heart Failure Questionnaire; NYHA: New York Heart Association; QOC: Quality Of Communication questionnaire; SF-36: Medical Outcomes Study 36-Item Short-Form Health Survey; SGRQ: St. Georges Respiratory Questionnaire;TUG: Timed 'Up and Go' test; VAS: Visual Analogue Scale; WALT: Willingness to Accept Life-sustaining Treatments.

## Competing interests

The authors declare that they have no competing interests that are directly relevant to the content of this article.

## Authors' contributions

DJAJ is responsible for study concept and design, drafting of the manuscript, critical revision of the manuscript and administrative, technical, or material support. EFMW is responsible for study concept and design, critical revision of the manuscript, obtained funding and study supervision. JMGAS is responsible for study concept and design, critical revision of the manuscript, obtained funding and study supervision. MAS is responsible for study concept and design, drafting of the manuscript, critical revision of the manuscript, obtained funding and study supervision. All authors read and approved the final manuscript.

## Pre-publication history

The pre-publication history for this paper can be accessed here:


